# Evaluation of Loopamp™ *Leishmania* Detection Kit and *Leishmania* Antigen ELISA for Post-Elimination Detection and Management of Visceral Leishmaniasis in Bangladesh

**DOI:** 10.3389/fcimb.2021.670759

**Published:** 2021-04-26

**Authors:** Faria Hossain, Albert Picado, Sophie I. Owen, Prakash Ghosh, Rajashree Chowdhury, Shomik Maruf, Md. Anik Ashfaq Khan, Md. Utba Rashid, Rupen Nath, James Baker, Debashis Ghosh, Emily R. Adams, Malcolm S. Duthie, Md. Sakhawat Hossain, Ariful Basher, Proggananda Nath, Fatima Aktar, Israel Cruz, Dinesh Mondal

**Affiliations:** ^1^ Emerging infections and Parasitology laboratory, Nutrition and Clinical Service Division, International Centre for Diarrhoeal Disease Research, Bangladesh, Dhaka, Bangladesh; ^2^ Neglected Tropical Diseases, Foundation for Innovative New Diagnostics, Geneva, Switzerland; ^3^ Department of Tropical Disease Biology, Liverpool School of Tropical Medicine, Liverpool, United Kingdom; ^4^ Faculty of Medicine, University of Leipzig, Leipzig, Germany; ^5^ Research, HDT Bio-Corp., Seattle, WA, United States; ^6^ National Kala-azar Elimination Program, CDC, DGHS, Dhaka, Bangladesh; ^7^ Department of Medicine, Infectious Disease Hospital, Dhaka, Bangladesh; ^8^ Infectious diseases and Tropical Medicine, Mymensingh Medical College and Hospital, Mymensingh, Bangladesh; ^9^ International Health Department, National School of Public Health, Instituto de Salud Carlos III, Madrid, Spain; ^10^ Laboratory Sciences and Services Division, International Centre for Diarrhoeal Disease Research, Bangladesh, Dhaka, Bangladesh

**Keywords:** visceral leishmaniasis, diagnosis, LAMP, qPCR, urine ELISA, elimination, diagnostics for VL post-elimination era

## Abstract

With reduced prevalence of visceral leishmaniasis (VL) in the Indian subcontinent (ISC), direct and field deployable diagnostic tests are needed to implement an effective diagnostic and surveillance algorithm for post-elimination VL control. In this regard, here we investigated the diagnostic efficacies of a loop-mediated isothermal amplification (LAMP) assay (Loopamp™ *Leishmania* Detection Kit, Eiken Chemical CO., Ltd, Japan), a real-time quantitative PCR assay (qPCR) and the *Leishmania* antigen ELISA (CLIN-TECH, UK) with different sampling techniques and evaluated their prospect to incorporate into post-elimination VL control strategies. Eighty clinically and rK39 rapid diagnostic test confirmed VL cases and 80 endemic healthy controls were enrolled in the study. Peripheral blood and dried blood spots (DBS) were collected from all the participants at the time of diagnosis. DNA was extracted from whole blood (WB) and DBS *via* silica columns (QIAGEN) and boil & spin (B&S) methods and tested with qPCR and Loopamp. Urine was collected from all participants at the time of diagnosis and was directly subjected to the *Leishmania* antigen ELISA. 41 patients were followed up and urine samples were collected at day 30 and day 180 after treatment and ELISA was performed. The sensitivities of the Loopamp-WB(B&S) and Loopamp-WB(QIA) were 96.2% (95% CI 89·43-99·22) and 95% (95% CI 87·69-98·62) respectively. The sensitivity of Loopamp-DBS(QIA) was 85% (95% CI 75·26- 92·00). The sensitivities of the qPCR-WB(QIA) and qPCR-DBS(QIA) were 93.8% (95% CI 86·01-97·94) and 72.5% (95% CI 61·38-81·90) respectively. The specificity of all molecular assays was 100%. The sensitivity and specificity of the *Leishmania* antigen ELISA were 97.5% (95% CI 91·47-99·70) and 91.95% (95% CI 84·12-96·70) respectively. The *Leishmania* antigen ELISA depicted clinical cure at day 180 in all the followed-up cases. Efficacy and sustainability identify the Loopamp-WB(B&S) and the *Leishmania* antigen ELISA as promising and minimally invasive VL diagnostic tools to support VL diagnostic and surveillance activities respectively in the post-elimination era.

## Introduction

Visceral leishmaniasis (VL), also known as kala-azar, is a vector-borne parasitic disease caused by protozoans of the *Leishmania donovani* complex, which are transmitted by phlebotomine sandflies (Chappuis et al., 2007). On the Indian subcontinent (ISC) the disease is caused by *Leishmania donovani*, which is transmitted in an anthroponotic cycle and is responsible for significant morbidity and mortality (Matlashewski et al., 2011). For years, the estimated global VL incidence ranged between 50,000-90,000 cases per year (Bi et al., 2018), and three countries, Bangladesh, India, and Nepal, contributed to 60-80% of the reported incidences^1^. In 2005, the three countries signed a memorandum of understanding and launched the kala-azar elimination program on the ISC to eliminate VL as a public health problem by 2015, later extended to 2020 (Selvapandiyan et al., 2019). The program aimed to attain less than 1 case per 10,000 people at the intervention unit level in three phases: achieving the target incidence in the attack phase, retaining the incidence for 3 consecutive years in the consolidation phase, and following validation by the World Health Organization (WHO), sustaining the target incidence in the maintenance phase (Le Rutte et al., 2018). Bangladesh and Nepal achieved the target in 2016 and 2013 respectively and wait for validation while incidence is reduced below the target in the majority of intervention units in India (Rijal et al., 2019). The attainment of the target incidence led to a drastic reduction in the reported case numbers in the three countries, from 41,158 in 2005 to 3,105 cases that contributed to a 77% decrease in global VL incidence in 2019^1^.

The reduction in VL prevalence on the ISC is attributed to the policies and strategies taken by the elimination program in the attack phase. Notably, the implementation of a point-of-care diagnostic test, the rK39 rapid diagnostic test (RDT), and an improved treatment regimen, single-dose liposomal amphotericin B (LAmB) accelerated the progression of the program (Olliaro et al., 2017). However, the program must re-evaluate detection strategies and interventions for the post-elimination era in accordance with a reduced VL prevalence. While the rK39 RDT acted as a protagonist during the attack phase, the assay is likely to lose its positive predictive value in the maintenance phase with fewer cases (Rijal et al., 2019) leading to the incorrect treatment of non-VL cases. Moreover, the rK39 RDT is unsuitable as a test of cure and for diagnosis of relapse cases due to the inability to differentiate past and new infection - an inherent limitation of indirect diagnostic approaches (Srividya et al., 2012). Furthermore, the existing diagnostic algorithm includes visible disease manifestations for at least two weeks (NKEP, CDC, 2016) which unfortunately extends the period of infectiousness and transmissibility. Therefore, a direct and field-deployable diagnostic method is crucial for prompt management of sporadic cases and limit the transmission in the post-elimination setting.

Despite sincere efforts, a sensitive, direct detection method that circumvents the limitations of the rK39 RDT and is suitable for peripheral settings remains unavailable. Molecular assays such as real-time quantitative polymerase chain reaction (qPCR) are the most sensitive direct detection methods; though, the high maintenance and associated cost confined its application to research relevant activities (Sundar and Singh, 2019). Persisting efforts led to the development of different sensitive molecular assays (e.g. Loop-mediated isothermal amplification-LAMP, recombinase polymerase amplification-RPA) that eliminate the necessity for expensive thermocyclers with shorter turnaround time, while utilization of dried reagents substantially increased the prospect of introducing the assays in near-patient settings (Mondal et al., 2016; Nzelu et al., 2019; Rijal et al., 2019). However, template preparation methods significantly influence the assay throughput and the requirement of expensive and labor-intensive nucleic acid extraction techniques often restrict such initiatives. Moreover, diagnostic techniques accompanying safer and non- or minimally invasive sampling methods are required to increase patient compliance, especially for the low prevalence setting in the post-elimination era.

In an endeavor to evaluate such direct diagnostic tools, in this study, we determined the efficacies of a reference qPCR and a LAMP assay (Loopamp™ *Leishmania* Detection Kit, Eiken Co., Ltd, Japan). The assay showed excellent efficacies in detecting VL cases in Sudan and cutaneous leishmaniasis cases in Afghanistan and Suriname previously (Mukhtar et al., 2018; Vink et al., 2018; Schallig et al., 2019). Here, we performed the Loopamp assay with whole blood and dried blood spots and evaluated the effect on the diagnostic efficacies of two different extraction methods, commercial silica-based spin-columns and an in-house boil & spin protocol. Furthermore, we also evaluated the *Leishmania* antigen ELISA (CLIN-TECH, UK) in urine samples, previously showing promise in the detection of VL cases from Bangladesh and Ethiopia in a pilot study (Vallur et al., 2015), as a non-invasive diagnostic assay and test of treatment response over 6 months.

## Methods and Materials

### Ethics Statement

The study was conducted in accordance with the Declaration of Helsinki and approved by the International Centre for Diarrheal Disease Research, Bangladesh (icddr,b) ethical review committee (PR-14093). Written informed consent was obtained from each adult participant or guardian of any participant aged less than 18 years.

### Study Sites and Participant Characteristics

All study participants were enrolled from Mymensingh division, a highly endemic region for VL that accounts for more than half of the total VL patients in Bangladesh. Sample collection and template preparation was performed at Surya Kanta Kala-azar Research Center (SKKRC), Mymensingh. Laboratory tests were performed at the icddr,b, Dhaka. All participants were enrolled between June 2016 to March 2018. Clinical diagnosis of the patients was performed according to the national guidelines of Bangladesh (NKEP, CDC, 2016). Individuals without a history of VL, suffering from a fever of more than two weeks in duration, splenomegaly, and positive with rK39 rapid detection test (RDT) were enrolled and defined as VL cases. Age and sex-matched clinically healthy household contacts of VL cases with no history of VL, or any symptoms of severe, acute, or chronic illness and a negative rK39 RDT were enrolled as endemic healthy controls. rK39 RDT was performed with serum sample of the suspected cases showing clinical symptoms by Kala-azar Detect™ Rapid Test kit according to the manufacturer’s instruction (Inbios International, Inc., USA). A total of 80 VL patients and 80 endemic healthy controls were included in the study. The admission, clinical management and treatment of patients was arranged at SKKRC. All VL patients were treated with a single dose intravenous infusion of 10 mg/kg LAmB, as per the national guidelines. Patients were followed up at 6 months and 12 months after treatment to monitor their response to treatment and development of VL related post-treatment complications.

### Clinical Specimens

Ten milliliters of whole blood and 50mL morning urine were collected from all participants at baseline. Serum was separated by centrifuging 3mL whole blood at a rate of 3,500 rpm for 5 minutes. Dried blood spots (DBS) were prepared by the addition of 45µL whole blood to Whatman^®^ FTA^®^ cards (Sigma-Aldrich). Urine was also collected from 41 VL patients at 30 and 180 days after completion of treatment. Cold chain (4°C) maintenance was assured while transporting the samples to icddr,b, for laboratory analysis.

### Template Preparation by QIAGEN Extraction Method

DNA was isolated from 200µL heparin treated whole blood (WB-QIA) and three 5mm punched-out circles from a dried blood spot (DBS-QIA) using a QIAamp DNA tissue and blood mini kit (QIAGEN, Hilden, Germany). Extracted DNA was eluted into 200μL and 150μL of elution buffer provided with the kits respectively, as per the manufacturer’s instructions. Extracted DNA samples were stored at –80°C.

### Template Preparation by Boil & Spin Method

Sixty microliters of heparin-treated whole blood was mixed with 60μl of extraction buffer (400 mM NaCl, 40 mM Tris pH 6·5, 0·4% SDS) by vortexing for 10 seconds. The suspension was then incubated in a heating block at 95°C for 5 minutes and centrifuged for 3 minutes at 10,000g. After centrifugation, 30μL of clear supernatant containing DNA extracted by boil & spin (WB-B&S) was transferred to a dilution tube containing 345μL of PCR grade water.

### qPCR

qPCR was performed with template DNA extracted by QIAGEN extraction method from whole blood and DBS, using a protocol described elsewhere, targeting the conserved REPL repeats of the *Leishmania* genome (Hossain et al., 2017). Briefly, 5µL template DNA, 10 µL of TaqMan^®^ Gene Expression Master Mix (Applied Biosystems), 1 µL pre-ordered Taqman primer-probe mix (Applied Biosystems), and PCR grade water were used to prepare 20 µL reaction mix. A Bio-Rad CFX96 iCycler system was utilized for amplification. The conditions for amplification were as follow- 10 min at 95°C, followed by 15 seconds at 95°C and 1 min at 60°C (45 cycles). A standard curve was generated in each run with 10 ng to 1 fg of parasite DNA extracted from *in vitro* cultured promastigotes (*L*. *donovani* MHOM/IN/80/DD8) corresponding to 10,000 to 0.1 parasites per reaction. One reaction with molecular grade water as a negative control in each assay. Samples with cycle threshold (Ct) > 40 were considered negative. Samples were analyzed in duplicate and for the case of an indeterminate result, an additional run was performed.

### Loopamp Assay

The Loopamp assay (Loopamp™ *Leishmania* Detection Kit, Eiken Chemical Co. Ltd, Japan) was performed according to the manufacturer’s instructions using template DNA from whole blood prepared by QIAGEN and B&S extraction methods, and DBS DNA extracted by QIAGEN extraction method. Template DNA (3μL) extracted by the QIAGEN method was added to each tube, supplied as a string of 8 tubes with lyophilized Master Mix in the tube caps. The volume was made up to 30μL by the addition of 27μL Loopamp buffer. For the template DNA prepared by B&S method, 3 μL template DNA was used directly. Each run used one positive control and 30μL LAMP buffer as a negative control. After the addition of the template, tubes were closed, the string was turned upside down and shaken firmly. The string was placed cap-side down on the bench for 2 minutes for reconstitution of the dried Master Mix. Following reconstitution, the solution was spun down to the bottom of the tubes and the string was incubated in the Loopamp™ LF-160 incubator (Eiken Chemical Co. Ltd) at 65°C for 40 minutes, then 80°C for 5 minutes. After thermal incubation, the tube string was placed in the fluorescent unit, and samples that illuminated under blue LED light were considered positive. The results were read by two independent interpreters. Discordance in interpretation was resolved by the assessment of a third interpreter.

### Quality and Concentration of Extracted DNA

The quality and concentration of the QIAGEN and B&S extracted DNA from different samples were assessed with Thermo Scientific Nanodrop™ 2000 Spectrophotometer (Thermo Scientific, Hilden, Germany). DNA concentration was determined from the OD value at 260 nm and the quality was assessed by the ratio of OD values at 260nm and 280nm, considering the ratio of good quality DNA ranges between 1·8-2·0, following the standard protocol (Desjardins and Conklin, 2010).

### *Leishmania* Antigen ELISA

The *Leishmania* antigen ELISA was performed with urine samples according to the manufacturer’s instructions (CLIN-TECH, UK). Briefly, samples were diluted 400X for VL and 20X for controls with assay diluent and 100μL added in duplicate to flat-bottom 96-well microtiter plates pre-coated with sheep anti-leishmanial antibodies (CLIN-TECH, UK). Six antigen calibrators from 0 to 50 urinary antigen unit (UAU)/mL were added to each plate in duplicate to generate a standard curve. The plate was incubated for 30 minutes at 37°C. Following four washes, 100μL of working strength Tracer (sheep anti-*Leishmania* antibody labeled with peroxidase) was added into each well and incubated for 30 minutes at 37°C. After another wash sequel, 100μL TMB substrate solution was added to each well and the plate was incubated for 30 minutes at room temperature and the reaction was stopped with the addition of 100μL stop solution (0·5M HCl). Optical density (OD) was measured at 450nm and 620nm (ELx808, Biotek) within 30 minutes of the addition of stop solution. A four parametric logistic curve was constructed from all of the VL antigen calibrator points using Gen 5 software. The assay was considered effective when the OD for the 50 UAU/mL standard was more than 1·5 and the OD for the 0 UAU/mL calibrator was less than 0·1. The concentrations of the samples were adjusted by correcting for the dilution factor. A receiver operating characteristic (ROC) curve was generated to set the cut-off concentration for positivity (supplementary information). Samples with concentration <3·11 UAU/mL were considered negative.

### Statistical Analysis

Clinical sensitivity and specificity of the assays were measured against VL case definition according to the national guideline as a gold standard. Sensitivity and specificity [with 95% confidence interval (CI)] were calculated using exact binomial methods for proportions. McNemar’s test was performed to evaluate discordance between the clinical evaluation and the assays. To evaluate the inter-assay discordance McNemar’s test and Cochran’s Q test were performed. A two-tailed paired t-test was performed to measure differences between the means of quantitative variables. A p-value < 0.05 was considered to indicate statistically significant differences. The relationship between the parasite loads detected by qPCR between WB and DBS variables was determined by the Pearson correlation coefficient. To measure the inter-assay agreement, the Cohen’s kappa statistic (k) was performed. The values of Cohen’s k coefficients were interpreted according to Landis and Koch: 1·00–0·81: excellent; 0·80–0·61: good; 0·60–0·41: moderate; 0·40–0·21: weak; and 0·20–0·00: negligible agreement (19). All statistical analyses were performed in SPSS 20 and Graphpad Prism 8·0.

## Results

### Patient Demographics

The clinical and demographic details of study participants are detailed in **Table 1**. All patients responded well to the treatment, were cured clinically by 6 months and none reported VL associated clinical complications in 12 months’ time after treatment.

**Table 1:** Clinical and demographic detail of study participants.

		VL cases (n=80)	Endemic healthy control (n=80)
Sex	Male	47 (58·8%)	46 (57·5%)
	Female	33 (41·2%)	34 (42·5%)
Age	5-11 years	17 (21·3%)	17 (21·3%)
	12-17 years	05 (6·3%)	03 (3·8%)
	18 ≥ years	58 (72·5%)	60 (75%)
Positive in rK39 RDT		80 (100%)	0 (0%)
Fever more than two weeks		80 (100%)	0 (0%)
Loss of appetite		50 (62·5%)	0 (0%)
Decreased body weight		25 (31·3%)	0 (0%)
Darkening of the skin		05 (6·3%)	0 (0%)
Bleeding from nose		02 (2·5%)	0 (0%)
Abdominal pain		16 (20%)	0 (0%)
Abdominal enlargement		17 (21·3%)	0 (0%)
Weakness		41 (51·2%)	0 (0%)
Pallor		58 (72·5%)	0 (0%)
Jaundice		06 (7·5%)	0 (0%)
Hepatomegaly		28 (35%)	0 (0%)
Splenomegaly		80 (100%)	0 (0%)

### Sensitivity, Specificity, and Comparative Analysis of the Assays

We evaluated an established qPCR assay, previously evaluated with buffy coat DNA, with WB and DBS samples. For the qPCR assay, the sensitivity of WB DNA extracted by QIAGEN extraction method was 93·8% (95% CI 86·01-97·94), whereas DBS DNA extracted by QIAGEN extraction method achieved a sensitivity of 72·5% (95% CI 61·38-81·90). The highest sensitivity by the Loopamp assay was achieved with WB-DNA extracted by boil & spin method at 96·2% (95% CI 89·43-99·22), followed by WB-DNA and DBS-DNA extracted by QIAGEN methods, achieving 95% (95% CI 87·69-98·62) and 85% (95% CI 75·26- 92·00) sensitivity, respectively. Both the assays achieved 100% specificity with every extraction method. Likewise, the *Leishmania* antigen ELISA achieved a 97·5% (95% CI 91·47-99·70) sensitivity that was highest among all the assays and a specificity of 91·9% (95% CI 84·12-96·70). There were no statistically significant differences in performance of qPCR-WB-QIA, Loopamp-WB-QIA, Loopamp-WB-B&S and *Leishmania* antigen ELISA to the clinical diagnosis of VL according to the national guideline, as determined by exact McNemar test (**Table 2**).

**Table 2:** Sensitivity and specificity of different assays along with P values in the McNemar test.

Assay	Sample type	Sensitivity (n/N)* (95% CI)	Specificity (n/N)¥ (95% CI)	p-value in Mc Nemar test
qPCR	WB-QIA	93·8 (75/80) (86·01-97·94)	100 (0/80) (95·49-100)	0·06
	DBS-QIA	72·5 (58/80) (61·38-81·90)	100 (0/80) (95·49-100)	0·00
Loopamp™ Leishmania Detection assay	WB-QIA	95·0 (76/80) (87·69-98·62)	100 (0/80) (95·49-100)	0·13
	WB-B&S	96·2 (77/80) (89·43-99·22)	100 (0/80) (95·49-100)	0·25
	DBS-QIA	85·0 (68/80) (75·26- 92·00)	100 (0/80) (95·49-100)	0·00
Leishmania antigen ELISA	Urine	97·5 (78/80) (91·47-99·70)	91·9 (73/80) (84·12-96·70)	0·180

*Number of positives out of the 80 true VL cases.

¥Number of positives out of the 80 endemic healthy controls.

**Figure 1:**
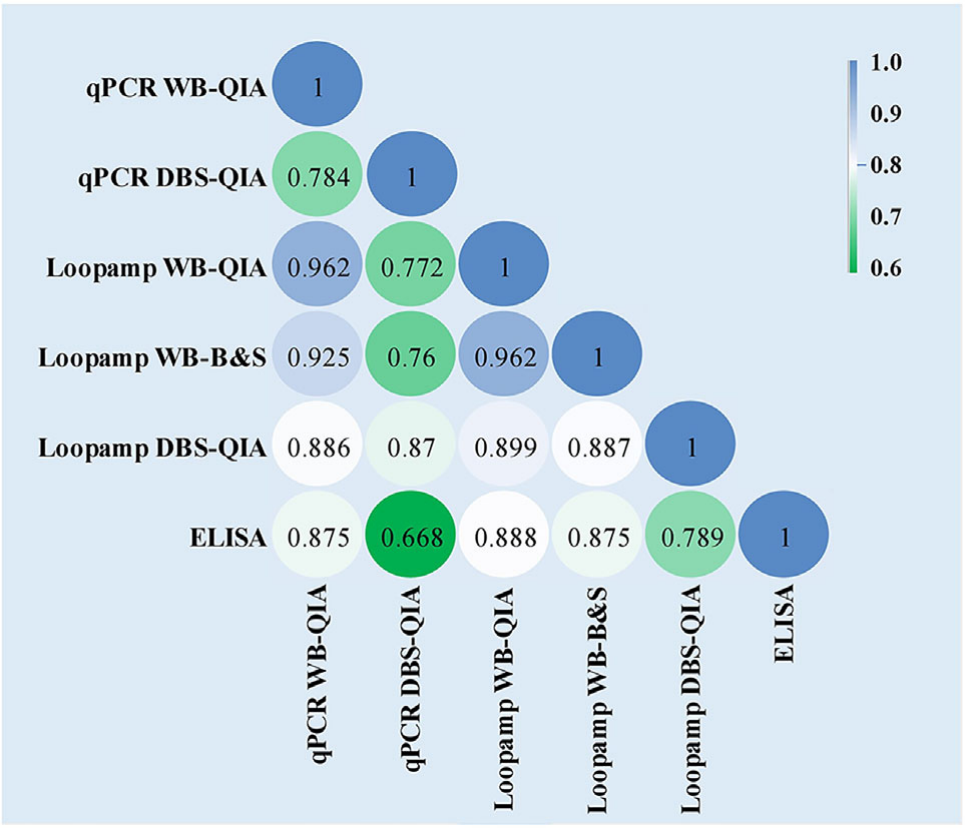
Inter-rater agreement observed between the assays presented as Cohen’s Kappa coefficients. The values of Cohen’s k coefficients are interpreted as: 1·00–0·81: excellent; 0·80–0·61: good; 0·60–0·41: moderate; 0·40–0·21: weak; and 0·20–0·00: negligible agreement.

No statistically significant difference was found in the overall sensitivities of the Loopamp and qPCR assays with whole blood samples. With DBS samples, sensitivity of Loopamp was significantly higher than qPCR. However, sensitivities of both Loopamp and qPCR were higher with whole blood than DBS samples. There were no statistically significant differences between the sensitivities of Loopamp/qPCR with whole blood and the urinary *Leishmania* antigen ELISA, however, the molecular assays performed significantly better in terms of specificity than the antigen ELISA (**Table 3**). The agreement between assays is shown in **Figure 1**.

**Table 3:** Comparison of different assays and samples on overall diagnostic performance.

Sample type	Assays	p-value (cases)	p-value (controls)	Overall performance
Whole blood	qPCR-QIA X Loopamp-QIA X Loopamp-B&S^a^	0·607	N/A	Equivalent
DBS	qPCR-QIA X Loopamp^b^-QIA	0·002	N/A	DBS: Loopamp-QIA**> qPCR-QIA
Whole blood X DBS	qPCR-QIA X Loopamp-QIA X Loopamp-B&SX qPCR-QIA X Loopamp-QIA^a^	0·000	N/A	WB: qPCR-QIA/Loopamp-QIA/Loopamp-B&S***> DBS: Loopamp-QIA**> DBS: qPCR-QIA
Whole blood X Urine	qPCR-QIA X Loopamp-QIA X Loopamp-B&S X Antigen ELISA^a^	0·392	0·000	WB: qPCR-Q/Loopamp-Q/Loopamp-B&S***> Urine: Antigen ELISA

^a^Cochran’s Q Test, ^b^ McNemar Test, N/A, Not analyzed due to constant values.

*p-value ≤0·05, **p-value < 0·01, ***p-value < 0·001.

### Effect of Extraction and Sampling Methods on Molecular Assays

**Figure 2:**
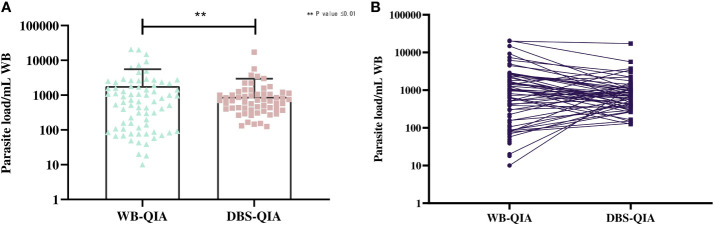
Differences in the parasite burden by qPCR between whole blood and DBS DNA extracted by QIAGEN method. **(A)** The mean parasite load in WB-QIA was 1,818 parasite/mL WB (SD=3,740 parasite/mL) and DBS-QIA was 899 parasite/mL WB (SD=2,069 parasite/mL). In a paired sample t-test, significant differences were observed in the mean parasite load between the two sample categories with t (79) =3·092, P=0·0027. **(B)** Trend in parasite load between the two sampling methods. Each point indicates the data obtained from an individual sample. The connecting line links data for each patient in WB-QIA and DBS-QIA method.

The parasite burden estimated by qPCR varied considerably between WB-QIA and DBS-QIA samples (**Figure 2**). In 77·5% (62/80) of the VL cases, the parasite load was greater in WB-QIA samples. An increased parasite load was observed in 22·5% of VL cases (18/80) by DBS-QIA method. However, a significant correlation was found in parasite load detected by qPCR between the two methods (r=0·723, p<0·001).

**Figure 3:**
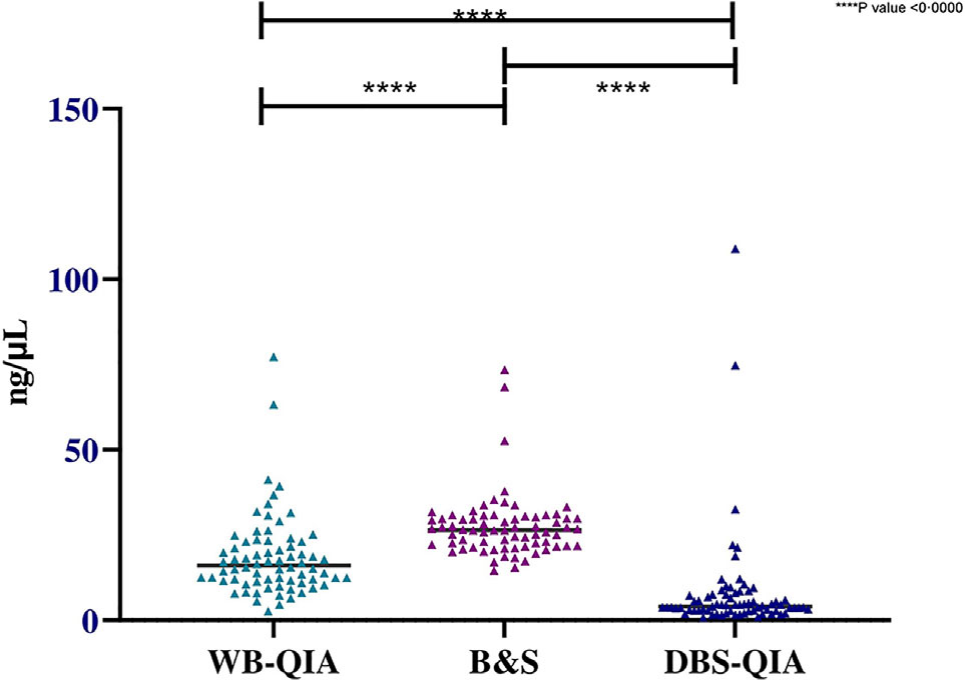
Concentration of DNA extracted by QIAGEN and boil & spin methods from whole blood and DBS samples(n=72). The mean DNA concentration was highest for WB-B&S, 27·51 ng/µL (SD=9·45 ng/µL), followed by WB-QIA and DBS-QIA, 18·68 ng/µL (SD=12·10 ng/µL) and 8·09 ng/µL (SD=15·47 ng/µL) respectively. In a two-tailed paired t-test, significant differences in the mean concentrations were observed for WB-QIA and WB-B&S (t(71)=4·981, P<0·0000), WB-B&S and DBS-QIA (t(71)=8·978, P<0·0000) and WB-Q and DBS-Q (t(71)=4·398, P<0·0000).

Significant variations in the mean DNA concentrations were observed for different sampling and DNA extraction methods when paired sample t-tests were performed (**Figure 3**). However, when considered quality, the mean 260/280 absorbance ratio were 1·829, 1·613 and 1·372 for WB-QIA, WB-B&S and DBS-QIA methods, respectively. The highest quality DNA was achieved by QIAGEN extraction method from whole blood, whereas WB-B&S and DBS-QIA the quality of the DNA was compromised by the presence of impurities.

### *Leishmania* Antigen Concentration as a Marker of Treatment Outcome

**Figure 4:**
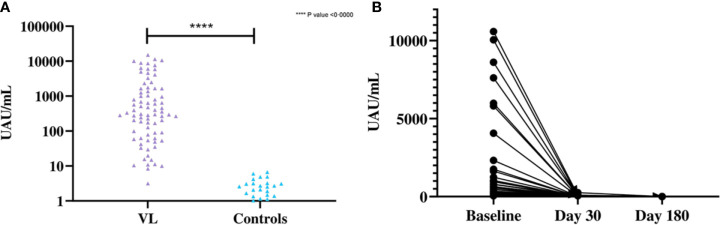
**(A)** Differences in the concentration of urinary antigen between VL and endemic control groups at baseline. The mean Ag concentration in the VL group was 1,706 UAU/mL (SD=3,106 UAU/mL) and the mean Ag concentration for controls was 0·8523 UAU/mL (SD=1·507 UAU/mL). Significant differences were observed by two-tailed t-test between the two groups (t (79) = 4·912, P<0·0001). **(B)** Concentration of urinary Ag in VL patients at baseline, 30 days after treatment and 180 days after treatment (n=41). The mean Ag concentration at baseline was 1,681 UAU/mL (SD=2,888 UAU/mL), 10·94 UAU/mL (SD=42·36 UAU/mL) at day 30 after treatment and 0·0585 UAU/mL (SD=0·375 UAU/mL) at day 180 after treatment.

Significant difference was observed in the mean antigen concentrations between the VL and control groups by *Leishmania* antigen ELISA (**Figure 4A**). The *Leishmania* antigen ELISA was also efficient in assessing treatment outcome. Of the 41 patients who provided samples post-treatment, 35 were antigen negative at day 30 and all patients were antigen negative at day 180 (**Figure 4B**).

### Comparative Assessment of the Assay Characteristic

The features of individual assays are summarized in **Table 4**. The time to result is inclusive of the time for sample processing and the kit cost includes only the reagent cost for sample processing (extraction etc.) and amplification.

**Table 4:** Comparison of assay characteristics.

	qPCR-WB-QIA	qPCR-DBS-QIA	Loopamp-WB-QIA	Loopamp-WB-B&S	Loopamp-DBS-QIA	Leishmania antigen ELISA
Detection	Real time, fluorescence	Real time, fluorescence	Endpoint, fluorescence	Endpoint, fluorescence	Endpoint, fluorescence	Endpoint, colorimetric
Sample preparation	Manual	Manual	Manual	Manual	Manual	Manual
Automation for detection and analysis	Yes	Yes	No	No	No	Yes
Assay validation	Quantitative	Quantitative	Qualitative	Qualitative	Qualitative	Quantitative
Detection of *L. donovani* markers	Direct	Direct	Direct	Direct	Direct	Direct
Mode of sample collection	Invasive	Invasive	Invasive	Invasive	Invasive	Non-invasive
Volume of sample required	200uL peripheral blood	200uL peripheral blood	200uL peripheral blood	60 uL peripheral blood	120 uL peripheral blood	5-10uL urine
Samples analyzed per assay	82	82	14	14	14	82
Time to result/assay	2:45 hours	3 hours	1:20 hour	1 hour	2:40 hours	2 hours
Kit cost/reaction	~16·5 USD	~18·9 USD	~10·12 USD	~6·92 USD	~12 USD	~6·12 USD
Instrument/cost/life expectancy	Bio-Rad CFX96 iCycler ~19,000 USD/15years	Bio-Rad CFX96 iCycler ~19,000 USD/15 years	Loopamp™ LF-160 incubator (Eiken Chemical Co.Ltd)/6000 USD/10 years	Loopamp™ LF-160 incubator (Eiken Chemical Co. Ltd)/6000 USD/10 years	Loopamp™ LF-160 incubator (Eiken Chemical Co.Ltd)/6000 USD/10 years	ELx808 ELISA reader (Biotek)/5000 USD/10 years
Storage temperature of reagents	-20°C	-20°C	Room temperature	Room temperature	Room temperature	4°C
Health care setting	Tertiary	Tertiary	Point-of-care	Point-of-care	Point-of-care	Secondary/Tertiary

## Discussion

Here, we performed the Loopamp assay with whole blood and DBS sampling with QIAGEN and B&S nucleic acid extraction methods and compared it with a reference qPCR assay under similar conditions, to determine the most appropriate combination of sampling-extraction-detection for clinical diagnosis of VL. The qPCR assay was assessed previously with buffy coat DNA from VL patients with promising efficacy (Hossain et al., 2017). However, detection of the parasite in the peripheral blood sample is preferred over buffy coat, as only 200µL instead of 1·5mL whole blood is required to prepare the same volume of template DNA. Besides, though the purity is low, the B&S method is faster and retains a sufficient concentration of template DNA (Barbosa et al., 2016).

In this study, the Loopamp-WB(B&S) achieved the greatest sensitivity (96·2%) among the molecular assays. The higher sensitivity of the Loopamp-WB(B&S) is attributed to the highest template DNA recovery, though the overall purity of the DNA is reduced relative to other extraction methods. In another study performed in Sudan, Loopamp-WB(B&S) achieved 97·6% sensitivity that is comparable to the present study, with the sensitivity of Loopamp-WB(QIA) was 100% compared to 95% sensitivity achieved here (Mukhtar et al., 2018). However, the qPCR assays showed lower sensitivities than the previous study with buffy coat-DNA (100%), suggesting the qPCR assay is more effective when performed with the buffy coat (Hossain et al., 2017).

The Loopamp assay showed higher sensitivity than qPCR in all combinations of sampling-extraction methods. The results varied from another study where a qPCR-WB(QIA) assay was more sensitive (96·1%) than the Loopamp-WB(QIA) (92·3%). However, the study incorporated qPCR targeting the kDNA and turbidity based LAMP product detection system, compared to the REPL repeat targeted qPCR assay and fluorimetric LAMP product detection in the present study (Adams et al., 2018). Moreover, the Loopamp assay offers high analytical sensitivity (10^-3^ parasite equivalents/reaction) and targets two different regions (*18SrRNA* gene and kDNA minicircles) (Adams et al., 2018; Ibarra-Meneses et al., 2018), whereas the analytical sensitivity of the qPCR is 10^-1^ parasite equivalent/reaction and a single target (Hossain et al., 2017). However, the efficacies of all three molecular assays with whole blood (Loopamp-B&S, Loopamp-QIA and qPCR-QIA) were statistically similar and both the Loopamp-WB(B&S) and Loopamp-WB(QIA) satisfy the required sensitivity and specificity (≥95% and ≥98% respectively) of an ideal diagnostic assay for VL case detection (Boelaert et al., 2007). The qPCR-WB(B&S) assay was found ineffective in our study (data are not shown), indicating interference by impurities (e.g., hemoglobin, protein antibodies etc), in agreement with previous studies (Sriworarat et al., 2015).

Use of a DBS from capillary blood collected by finger-prick would further reduce the invasiveness of sampling, eliminate the need for cold-chain during transportation, and would be appropriate for mass sample collection during surveillance. PCR experiments with DBS previously reported variable sensitivities (70% and 90%) (Sundar and Singh, 2019). However, to our knowledge, this is the first study evaluating DBS samples in both qPCR and Loopamp assays for *L.donovani* detection. In our study, the Loopamp-DBS(QIA) and qPCR-DBS(QIA) assays were moderately sensitive, and the Loopamp assay yielded better results than qPCR. These lower sensitivities in qPCR and Loopamp assay with DBS samples is also documented for conventional PCR (Smit et al., 2014) and infers inadequate template availability, as substantiated with the observed loss in template DNA recovery and reduced parasite load in DBS samples. Moreover, both Loopamp-DBS(B&S) and qPCR-DBS(B&S) assays were unable to detect parasite DNA (data not shown), depicting the necessity of additional processing and purification steps for the B&S method.

Integration of a test of cure and treatment monitoring, potentially based on an antigen-based, non-invasive diagnosis will benefit the post-elimination program. VL accompanies renal dysfunction and associated nephropathy, resulting in the excretion of the parasite antigens in the urine (Bezerra et al., 2019). Therefore, detection of the *Leishmania* antigens in urine for VL diagnosis is a promising non-invasive diagnostic approach to support the post-elimination program. We investigated one such prospective urine-based detection system, the *Leishmania* antigen ELISA. It is a quantitative, capture ELISA exploiting anti-*Leishmania* poly-clonal antibodies produced against whole promastigotes to detect *Leishmania* antigens excreted in urine (Vallur et al., 2015). The sensitivity in this study was 97·5% with significant differences in antigen concentration between the case and controls. However, the specificity in the endemic controls was moderate, consistent with our previous study (90%), however, we set our cut-off concentration from receiver operating characteristic (ROC) curve to adjust the precision (Vallur et al., 2015). The assay performance was statistically inferior in terms of specificity when tested against the molecular assays of similar sensitivities. However, the assay was accurate in conferring complete clinical cure at day 180, in agreement with the definition of clinically cured cases according to the current national guideline, (NKEP, CDC , 2016) and further assured by no reported clinical complications related to VL in a one-year follow up period. The results are consistent with a previous study in Ethiopian subjects with similar accuracy to determine clinical cure (Vallur et al., 2015).

Attaining the VL elimination status will subsequently limit funding and programmatic activities are likely to be committed to the public healthcare system. Therefore, apart from the efficacy, the aptness of the assays for the post-elimination setting hinges upon other parameters e.g., cost, feasibility, scalability, and robustness. Reckoning the efficacy, feasibility, and cost, the Loopamp assay succeeded the qPCR assay in our study and emerged as a promising alternative (**Table 4**). Lyophilized reagents, one step-single tube LAMP assay, compact device, and visual detection of the LAMP products with the in-built fluorescence unit of the Loopamp™ LF-160 incubator made the assay apt for peripheral health care setting (Besuschio et al., 2017; Adams et al., 2018). Coupling the B&S extraction with the Loopamp assay further lessened the time and cost along with a simplified protocol that increased the assay feasibility. The assay is also minimally invasive and requires only 60µL of blood as an initial sample which is equivalent to 2 drops of peripheral blood. The sampling feasibility can be therefore increased utilizing finger-pricked blood and requires investigation. Additionally, the availability of the Loopamp TB (WHO recommended) and Malaria detection kit increases its prospect to be exploited as an integrated diagnostic platform along with VL (World Malaria Report, 2019, Geneva).

The limitations of the Loopamp assay comprise its limited throughput and absence of the quantification feature compared to the qPCR. Also, utilization of FTA cards in DBS-Loopamp increased the assay cost (**Table 4**), rendering the assay less desirable for mass screening during post-elimination surveillance. The *Leishmania* antigen ELISA in contrast is more compatible for surveillance studies as another sensitive, cost-effective, high-throughput, and non-invasive diagnostic tool, offering sampling feasibility and increased patient compliance (Abeijon and Campos-Neto, 2013). The prospect of the assay to identify asymptomatic carriers from a cohort is explored in a recent study with promising outcome (Owen et al., 2021). Its unique quantification feature can also be exploited to determine treatment outcome. However, the low specificity may cause inconclusive results, requiring a secondary test for confirmation. For basic laboratory research requiring precise quantification of parasites, e.g., evaluation of novel vaccines, drugs, and diagnostic innovations etc., however, the qPCR assay is preferred.

Defining elimination by prevalence instead of the transmission status is a caveat in the elimination strategy and the continuous transmission, as evidenced by increased non-endemic VL cases, is impeding the validation process (Olliaro et al., 2017; Cloots et al., 2020). Therefore, in light of other neglected tropical disease (NTD) elimination programs (e.g. filariasis and schistosomiasis), the VL endgame policies should consider the interruption of transmission as the basis of infection containment in the post-elimination era (Stothard et al., 2017; Fang and Zhang, 2019). Effective interruption of VL transmission can only be achieved by reducing the transmission window of the infection *via* early diagnosis. Therefore, the VL patients according to the national guidelines that is the presence of clinical symptoms for two weeks along with rK39 positivity (NKEP, CDC, 2016). The enrollment was completed at SKKRC which is a referral center for VL, and the suspects were primarily diagnosed elsewhere. Therefore, all the suspects were clinically advanced and rK39 RDT positive during enrollment. As such, the efficacies of the assays for VL detection earlier than two weeks remains unknown and further investigation is required with stratified time points since the onset of the symptoms. The microscopic detection of parasites in splenic aspirate was initially included as a diagnostic gold standard for VL in the study protocol. However, the procedure was discontinued in the early phases of the study following a serious adverse event reported. Also, the performance of the Loopamp assay as a test-of-cure was not evaluated and the efficacies of both the assays to detect treatment failures and relapse cases requires investigation.

In absence of definite tools to measure VL transmission, identification of the infectious pockets *via* epidemiological surveillance and ensuring early diagnosis are the only reliable measures to contain transmission in the post-elimination setting. Our study presented the Loopamp™ *Leishmania* Detection Kit with B&S extracted whole blood DNA and the *Leishmania* antigen ELISA promising to facilitate such post elimination diagnosis and epidemiological surveillances, respectively. A phase-3 diagnostic trial is therefore recommended in the peripheral settings to integrate the assays into the post-elimination VL control strategies.

## Data Availability Statement

The original contributions presented in the study are included in the article/**Supplementary Material**. Further inquiries can be directed to the corresponding author.

## Ethics Statement

The studies involving human participants were reviewed and approved by Ethical Review Committee, icddr,b. Written informed consent to participate in this study was provided by the participants’ legal guardian/next of kin.

## Author Contributions

FH: data curation, formal analysis, investigation, project administration, resources, validation, visualization, writing – original draft, and writing – review & editing. AP: conceptualization, formal analysis, funding acquisition, methodology, resources, supervision, validation, visualization, writing – original draft, and writing – review & editing. SIO: formal analysis, resources, and writing – review & editing. PG: formal analysis, investigation, project administration, and writing – review & editing. RC: investigation, resources, and writing – review & editing. SM: project administration, resources, and writing – review & editing. MAAK: investigation, resources, and writing – review & editing. MUR: formal analysis, resources, and writing – review & editing. RN: investigation, resources, and writing – review & editing. JB: investigation, resources, and writing – review & editing. DG: project administration, resources, and writing – review & editing. ERA: funding acquisition, conceptualization, methodology, resources, and writing – review & editing. MSD: formal analysis, resources, and writing – review & editing. MSH: data curation, software, resources, and writing – review & editing. AB: investigation, resources, and writing – review & editing. PN: investigation, resources, and writing – review & editing. FA: investigation, resources, and writing – review & editing. IC: conceptualization, data curation, formal analysis, funding acquisition, methodology, project administration, resources, supervision, visualization, and writing – review & editing. DM: conceptualization, data curation, formal analysis, funding acquisition, methodology, project administration, resources, supervision, visualization, and writing – review & editing. All authors contributed to the article and approved the submitted version.

## Funding

This work was largely supported by funds from the Federal Ministry of Education and Research, Germany (KfW grant reference number 202060457, Development of Products for the Prevention, Diagnosis and Treatment of Neglected and Poverty Related Diseases; https://www.bmbf.de/en), Wellcome Trust Seed funding (grant no. 108080/Z/15/Z) and the MRC-DTP (grant no. MR/N013514/1). UK aid from the UK Government, the Government of Switzerland and the Government of Netherlands also contributed to FIND’s participation in this work.

## Conflict of Interest

Author MSD was employed by HDT Bio-Corp.

The remaining authors declare that the research was conducted in the absence of any commercial or financial relationships that could be construed as a potential conflict of interest.

## Footnotes

1 Global Health Observatory data repository, WHO, 2021. https://apps.who.int/gho/data/node.main.NTDLEISHVNUM [Accessed February 2021].

## Supplementary Material

Supplementary Figure 1 Receiver operating characteristic (ROC) curve for Leishmania Antigen ELISA for determination of the cut-off Urinary antigen unit (UAU) for the detection of Leishmania antigens in urine samples. From the ROC curve, the selected combination of the sensitivity and specificity was 97.50% and 91.25% which gave a cut-off of >3.11 UAU.

Supplementary Figure 2 Study activity flow diagram.

Supplementary Figure 3 Standards for Reporting of Diagnostic Accuracy (STARD) flow diagram. **(A)** Sample flow and test results for cases. **(B)** Sample flow and test results for controls.
