# Supplementary figures and images for: Evaluation of Loopamp™ Leishmania Detection Kit and Leishmania Antigen ELISA for Post-Elimination Detection and Management of Visceral Leishmaniasis in Bangladesh
Source: Front Cell Infect Microbiol. 2021 Apr 26;11:670759. doi: 10.3389/fcimb.2021.670759 (PMC8108992; doi:10.3389/fcimb.2021.670759)

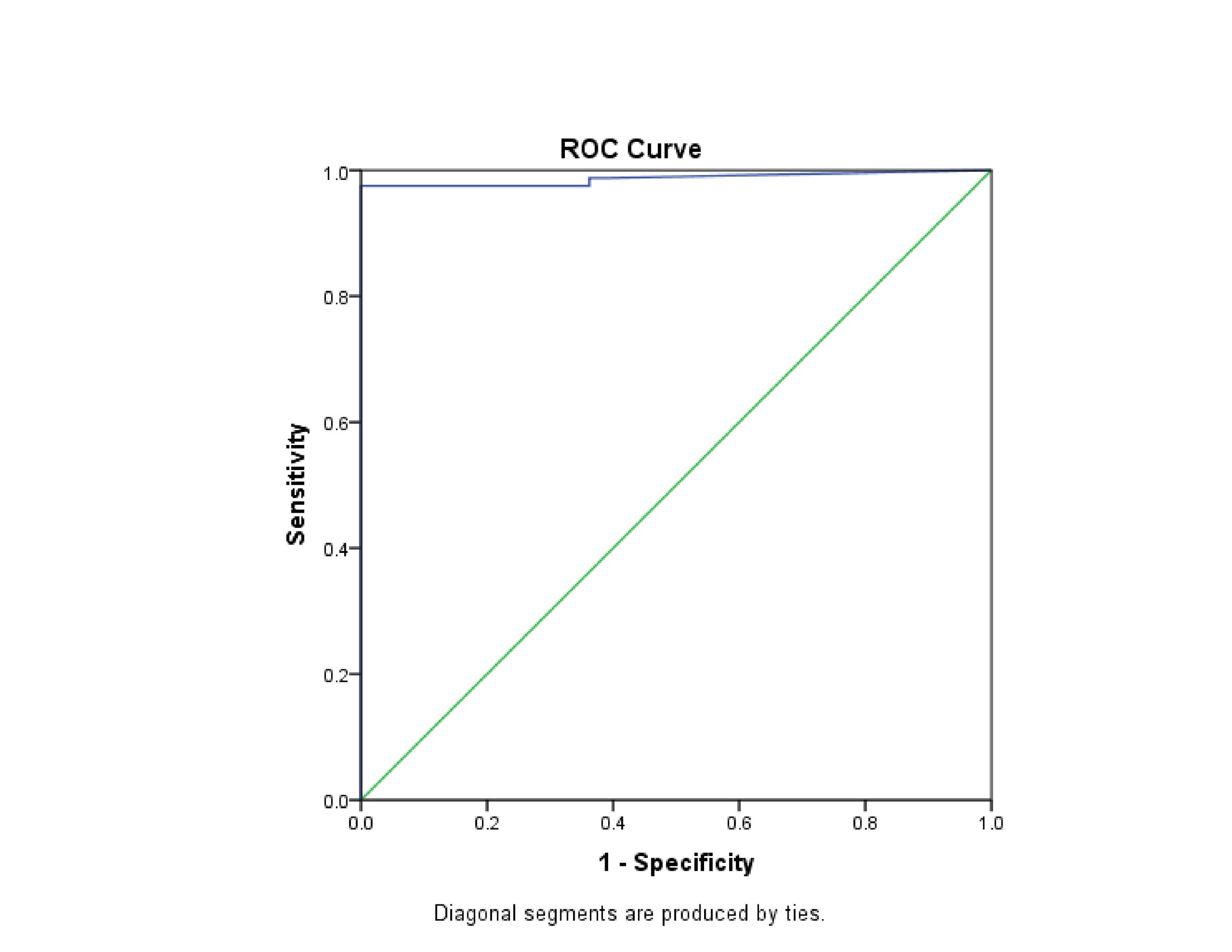

Supplement: Supplementary Figure 1 — Receiver operating characteristic (ROC) curve for Leishmania Antigen ELISA for determination of the cut-off Urinary antigen unit (UAU) for the detection of Leishmania antigens in urine samples. From the ROC curve, the selected combination of the sensitivity and specificity was 97.50% and 91.25% which gave a cut-off of >3.11 UAU. [file Image_1.jpeg]

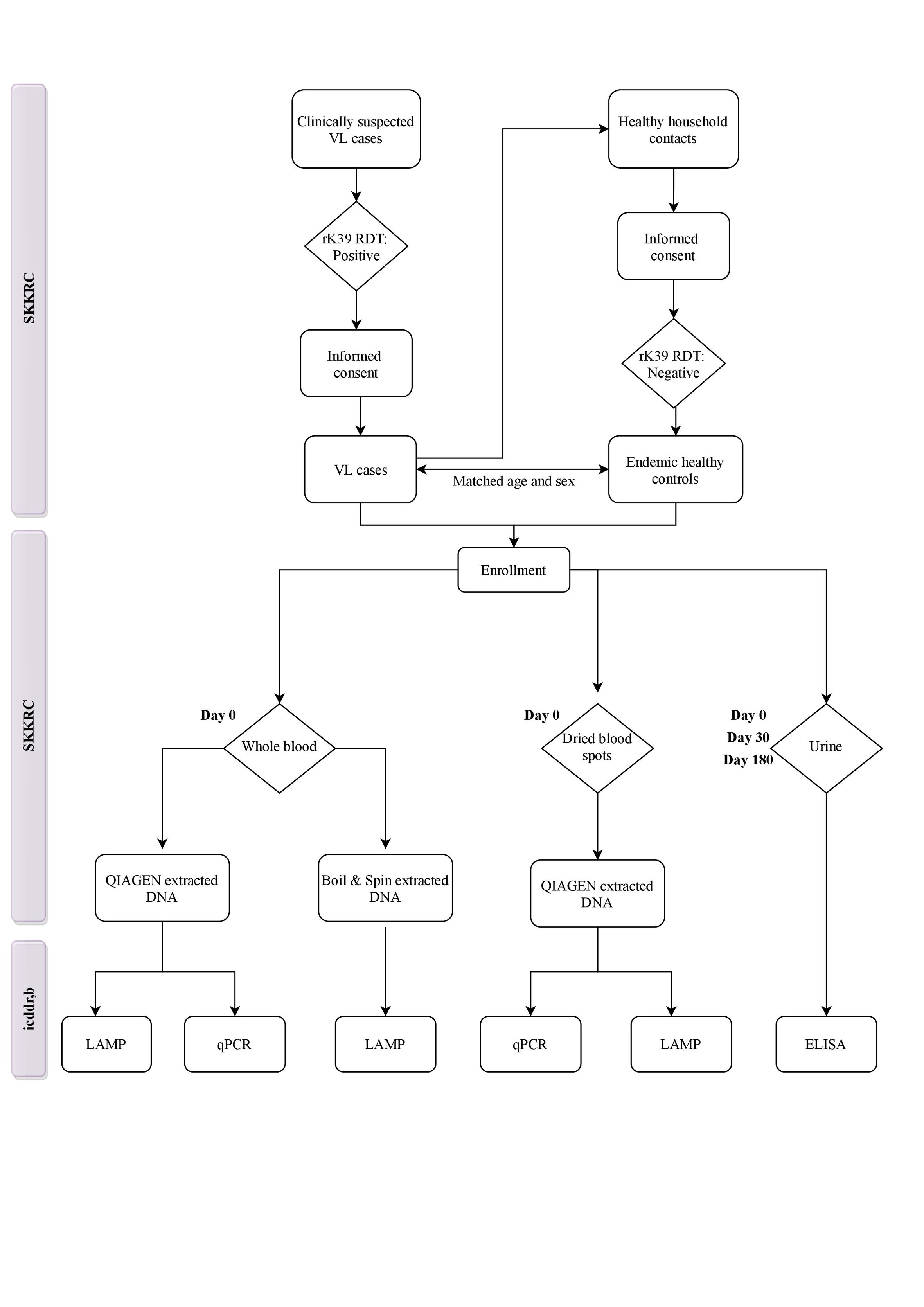

Supplement: Supplementary Figure 2 — Study activity flow diagram. [file Image_2.jpeg]

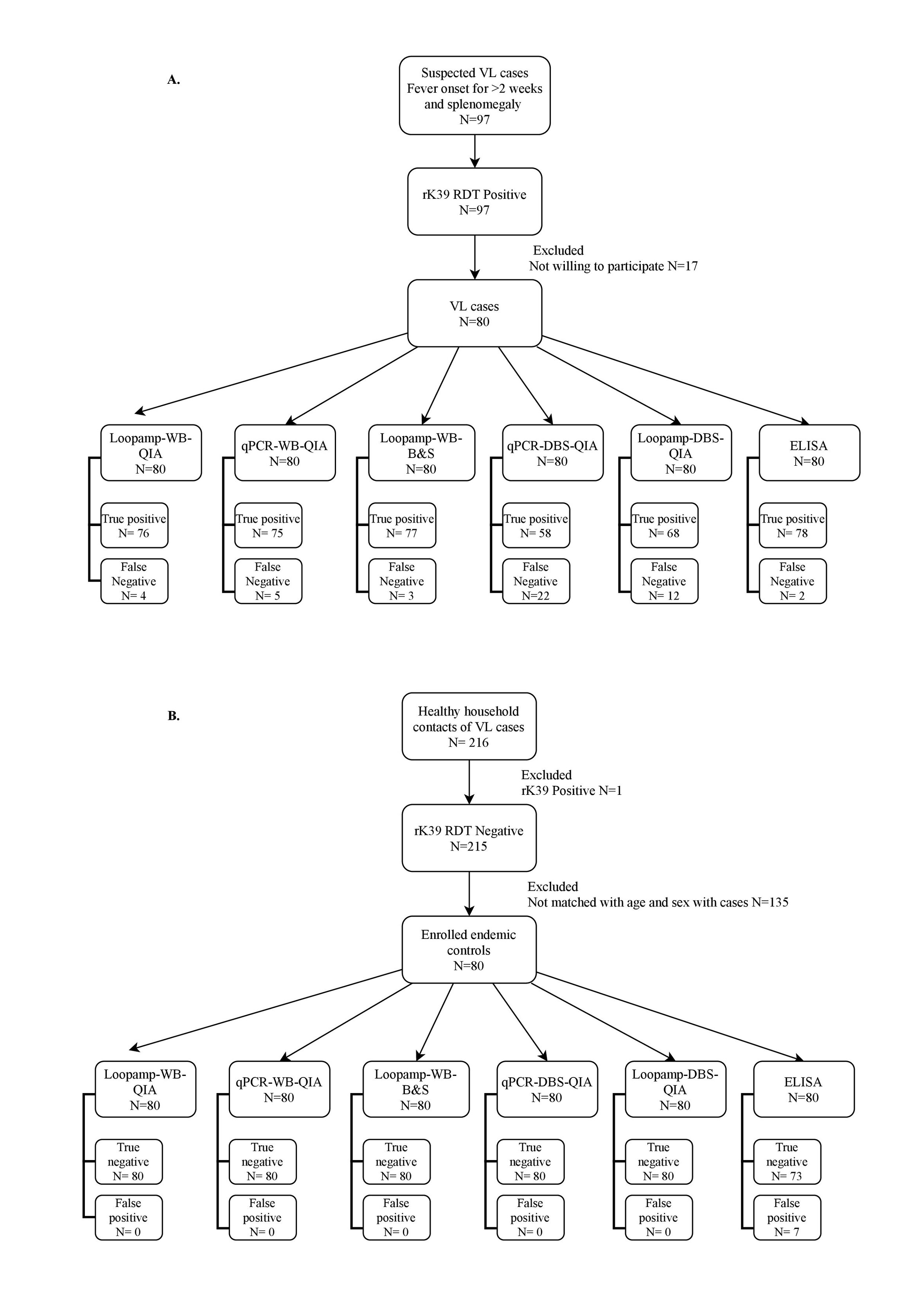

Supplement: Supplementary Figure 3 — Standards for Reporting of Diagnostic Accuracy (STARD) flow diagram. (A) Sample flow and test results for cases. (B) Sample flow and test results for controls. [file Image_3.jpeg]
